# *Stilbocrea banihashemiana* sp. nov. a New Fungal Pathogen Causing Stem Cankers and Twig Dieback of Fruit Trees

**DOI:** 10.3390/jof8070694

**Published:** 2022-06-30

**Authors:** Zeinab Bolboli, Behnaz Tavakolian, Reza Mostowfizadeh-Ghalamfarsa, Moslem Jafari, Santa Olga Cacciola

**Affiliations:** 1 Department of Plant Protection, School of Agriculture, Shiraz University, Shiraz 7144113131, Iran; bolbolizeinab@gmail.com (Z.B.); b.tavakolian72@gmail.com (B.T.); 2 Fig Research Station, Fars Agricultural and Natural Resources Research and Education Center, Agricultural Research, Education and Extension Organization (AREEO), Estahban 7451877802, Iran; m.jafary@areeo.ac.ir; 3 Department of Agriculture, Food and Environment (Di3A), University of Catania, 95123 Catania, Italy

**Keywords:** ascomycetous fungi, *Bionectriaceae*, *Eriobotrya japonica*, *Ficus carica*, ITS, multigene phylogenetic analysis, new taxon, *rpb2*, *tef1*

## Abstract

Stem cankers and twig dieback were the most serious disease of fig (*Ficus carica*) and loquat (*Eriobotrya japonica*) noticed in a survey of fruit tree orchards in the Fars Province, Iran. Isolates of *Bionectriaceae* were consistently recovered from symptomatic fig and loquat trees. Phylogenetic analyses of multiple nuclear loci, internal transcribed spacer regions (ITS) of rDNA, RNA polymerase II subunit 2 (*rpb2*), and translation elongation factor 1-α (*tef1*), combined with morphological observations, revealed that isolates could be referred to a still unknown taxon, which was formally described as *Stilbocrea banihashemiana* sp. nov. Phylogenetically, isolates from fig and loquat trees clustered in a well-supported monophyletic group within the *Stilbocrea* clade of *Bionectriaceae*, closely related to *S. walteri*. *Stilbocrea banihashemiana* sp. nov. was characterized by the lack of stilbella-like asexual structure in both natural substrates and pure cultures and produced two morphologically distinct types of conidia, globose and cylindrical, formed on short and long simple phialides. In pathogenicity tests, *S. banihashemiana* sp. nov. induced stem cankers in both fig and loquat, wood discoloration in fig and twig dieback in loquat. Pathogenicity tests also showed that the potential host range of this novel pathogen includes other economically relevant horticultural trees.

## 1. Introduction

The edible or common fig (*Ficus carica* L., family *Moraceae*) is a deciduous species native to southwest Asia and the Mediterranean region and the loquat or Japanese medlar [*Eriobotrya japonica* (Thunb.) Lindl., family *Rosaceae*] is an evergreen species supposed to be native to China and has also grown since ancient times in southern Japan; they are adapted to subtropical to temperate climates and are widely grown worldwide. Both are fruit trees of economic relevance in Iran. The Smyrna-type fig, including the two major groups of dried and fresh figs, is one of the most important horticultural crops in Iran, with 51,000 ha in arid and semi-arid regions of the country devoted to the cultivation of this fruit tree [1,2]. Iran is the fourth largest fig producer country in the world, after Turkey, Morocco, and Greece, and produces annually 56,557 tons of figs [3]. About 90% of fig-cultivations area in Iran is located in Fars Province. This region is also the third loquat producer in Iran, with an annual production capacity of 432 tons [4].

Stem cankers and twig dieback are one of the most serious diseases of fig and loquat worldwide. The casual agents of these diseases belong to different families of ascomycetous fungi, including *Diaporthaceae* [5], *Nectriaceae*, *Botryosphaeriaceae* [6,7,8,9], and *Ceratocystidaceae* [10]. These pathogens cause almost indistinguishable symptoms. In a recent survey aimed to identify ascomycetous pathogens associated with a widespread decline of fruit orchards in southern Iran, *Bionectriaceae* isolates were the fungi recovered most frequently from fig and loquat trees with symptoms of stem cankers and twig dieback in Fars Province.

Members of *Bionectriaceae* are less known as canker pathogens. However, also this family encompasses species that were reported as causal agents of cankers on woody plants, such as *Nectriella pironii* Alfieri & Samuels [11], which causes gall and stem cankers on fig, and *Geosmithia morbida* M. Kolark, E. Freeland, C. Utley & Tissera, the causal agent of Thousand Cankers Disease (TCD) of walnut (*Juglans regia* L.) [12,13,14]. Moreover, some species of *Stilbocrea*, a genus within this family, cause wood discoloration and trunk diseases, e.g., *S. colubrensis* Lechat & J. Fourn on *Bambusa vulgaris* (Schrad. ex J.C. Wendl.) Nakai [15] and *S. walteri* on *Quercus ilex* L. [16] and diverse *Citrus* species, including *Citrus aurantifolia* (Christm.) Swingle, *C. aurantium* L., and *C. limon* (L.) Osbeck [17]. *Stilbocrea macrostoma* (Berk. &. M.A. Curtis) Höhn was reported to be responsible for wood necrosis and decline symptoms on oak (*Quercus brantii* Lindl.) trees in Iran [18].

In this study, *Bionectriaceae* isolates, recovered from fig and loquat trees with symptoms of stem cankers and twig dieback in the Fars Province (southern Iran), were identified as a new species of *Stilbocrea* and their pathogenicity was evaluated to fulfill Koch’s postulates and to determine if they were able to infect other fruit trees of economic importance in Iran.

## 2. Materials and Methods

### 2.1. Sampling and Fungal Isolation

Fig and loquat orchards in several counties (Estahban, Darab, Firuzabad, Jahrom, Kazerun, Khafr, Neyriz, and Shiraz) of Fars Province, southern Iran, were surveyed during the tree dormancy period for three consecutive years (2019–2021). Trees of different commercial fig cultivars, including ‘Sabz’ and ‘Payves’ among dry figs, ‘Shah Anjeer,’ ‘Siah,’ and ‘Bargchenary’ among fresh figs, and ‘Pouzdonbali’ among caprifigs, as well as loquat trees, were sampled. Trunks, branches, and shoots with symptoms of canker, dieback, and decline were collected. Isolations were performed from transverse sections of symptomatic stems and twigs. Small tissue pieces (5 × 5 mm) from the margins between healthy and discolored wood were picked up with a scalpel, washed under running tap water, disinfected for 1 min in 70% ethanol, 1 min in a 2% sodium hypochlorite solution, and subsequently rinsed twice in sterile distilled water [8]. The disinfected segments were air-dried on a sterile paper towel for 10 min and then plated in Petri dishes containing potato dextrose agar (PDA, extract of 300 g/L boiled potato, 20 g glucose monohydrate, 15 g/L agar, distilled water) amended with tetracycline (1 mg/L). Plates were incubated at 25 °C for seven days. Emerging colonies were transferred into water agar (WA, 20 g/L agar, distilled water) and pure cultures were obtained by picking up single hypha tips after three days of incubation. 

### 2.2. Morphological Characterization

All *Bionectriaceae* isolates were identified and characterized according to Voglmyr & Jaklitsch [16]. For the colony morphology characterization, mycelial plugs from each isolate were placed on PDA and malt extract agar (MEA, 20 g/L of malt extract, 15 g/L agar, distilled water) and incubated at 25 °C with approximately 12 h of daylight and 12 h of darkness. Identification of isolates was based on colony morphology, pigment production on PDA, microscopic structures including conidiophore morphology, phialide shape and type, and conidial shape and size. Fungal structures were mounted in lactic acid, and 30 conidia and other fungal structures were measured. Temperature-growth relationships and average growth rates were tested on PDA and MEA (80 mm Petri dishes with 25 mL media) measured after 14 days of incubation under 12-h photoperiod with three replicate dishes per isolate and incubated at 5, 10, 15, 20, 25, 30, and 35 °C [16]. Isolates with the same morphological and cultural characteristics were grouped and representative isolates were further analyzed.

### 2.3. DNA Extraction, PCR Amplification, and Sequencing

For DNA extraction, representative isolates were cultured in potato broth (PB, extract of 300 g/L boiled potato in distilled water) for 10–15 days. DNA was extracted from harvested and freeze-dried mycelium using the DNG-PLUS extraction kit (CinnaGen, Tehran, Iran) by following the instructions provided by Mirsoleimani and Mostowfizadeh-Ghalamfarsa [19]. The quality and quantity of the extracted DNA were assessed through an MD-1000 Nanodrop spectrophotometer (NanoDrop Technologies, Wilmington, DE, USA). Primer pairs ITS1/ITS4 [20], EF1-728F [21] /EF1-2218R [22], and fRPB2-5F/fRPB2-7cR [23] were used to amplify nuclear ribosomal DNA internal transcribed spacer (ITS) region, translation elongation factor 1-α (*tef1*) and RNA polymerase II subunit 2 (*rpb2*), respectively. Polymerase chain reaction (PCR) mixtures were made in 25 μL volumes, consisting of 1 μL genomic DNA (~100 ng), 1 μL forward and reverse primers (10 pM), and 12.5 μL Taq DNA Polymerase 2× Master Mix RED (Amplicon, Odense, Denmark), and 9.5 μL PCR quality water. The annealing temperatures and time conditions used for PCR amplification are shown in detail in Table 1. PCR amplifications were performed on a Peltier Thermal Cycler (Bio-Techne, Minneapolis, MN, USA). PCR products were sequenced with the primers used for amplification by a dye terminator cycle (Cardiogenetic Research Center, Tehran, Iran). Sequences, initially identified using the BLAST approach [24], were deposited into GenBank [25].

### 2.4. Phylogenetic Analyses

New sequences generated in this study were edited, proofread, and concatenated in BioEdit v. 7.0.9.0 [26]. They were blasted against the NCBI’s GenBank nucleotide database to obtain highly similar sequences to representative isolates and preliminary identifications for phylogenetic inferences. The sequences generated in this study were aligned with the downloaded GenBank sequences by Clustal X [27] with subsequent visual adjustment. Partition homogeneity tests were conducted on combined nuclear gene alignment by PAUP* 4.0a136 [28] using 100 replicates and the heuristic general search option. Bayesian inference analyses on individual and concatenated ITS, *rpb2*, and *tef1* loci were carried out to reconstruct the phylogenetic trees, using MrBayes 3.1 [29]. The phylogenetic analysis of the *Bionectriaceae* dataset included 35, 21, 16, and 15 ingroup taxa for individual gene ITS, *rpb2*, *tef1*, and their concatenated combination, respectively. Two *Nectriaceae* members, including *Nectria cinnabarina* (Tode) Fr. strain AR4477 and *Thyronectria rhodochlora* (Mont.) Seeler strain NP2 were used as outgroup taxa in all phylogenetic trees (Supplementary Table S1). Estimation of the best-fit nucleotide substitution model was determined by MrModelTest 2.3 [30]. Two independent Markov chain Monte Carlo (MCMC) runs using four chains were run over 1,000,000 generations. Trees were saved each 1000 generations, resulting in 10,001 trees. Burn-in was set at 25% generations. To conduct a phylogenetic comparison, maximum likelihood (ML) estimation was carried out using PHYLIP DNAML [31] with the same data set. Evaluation of the robustness of the maximum likelihood trees was conducted by the bootstrapping method with 1000 replicates. TreeGraph was used for editing and displaying phylogenetic trees [32]. Alignments and trees were submitted to TreeBASE [33].

### 2.5. Pathogenicity Tests

A preliminary pathogenicity evaluation of representative isolates was conducted on detached shoots (5–9 mm in diameter) collected from 5- to 12-year-old fig and loquat trees that were cut into pieces of 25–30 cm in length. A pathogenicity test was also carried out on mature one-year-old fig ‘Sabz’ (the most widely grown commercial cultivar) and loquat saplings in greenhouse conditions at 26 ± 3 °C. Saplings were inoculated in February 2020. To determine the potential host range of representative isolates, additional pathogenicity tests were performed on detached stems of other fruit trees, including almond, olive, apple, quince, and pomegranate, that were adjacent to the fig and loquat trees in surveyed orchards. In all pathogenicity tests, the stem surface was cleaned and disinfested with 70% ethanol. The inoculation site was wounded using a 6-mm sterilized cork borer to remove the bark. A 6-mm diameter disc was then taken from the margin of a seven-day-old culture on PDA, inserted into the wound, covered with the bark disk, and sealed with Parafilm^®^ (Bemis Packaging, Sheboygan Falls, WI, USA) to prevent contamination. Non-colonized PDA agar plugs were used for the negative controls [34]. Both inoculated and control shoots and stems were put into glass bottles containing 100 mL of sterilized water and kept under greenhouse conditions at 25 ± 2 °C. Experiments were arranged according to a completely randomized design. On detached shoots and stems, symptoms were recorded 21 days post-inoculation (dpi) by gently removing the bark and measuring the lesion length. Inoculated saplings and their respective controls were taken back to the laboratory 30 dpi, the bark was removed, and symptoms severity was rated by measuring the lesion length. For re-isolations from artificially inoculated stems and saplings, five pieces (2 × 5 mm) of necrotic tissue from the edge of each lesion were cut, surface-disinfected [8], and placed on PDA in an attempt to re-isolate the inoculated fungi and complete Koch’s postulates. The isolates were identified based on morphological characteristics.

## 3. Results

### 3.1. Field Surveys and Disease Symptoms

**Figure 1:**
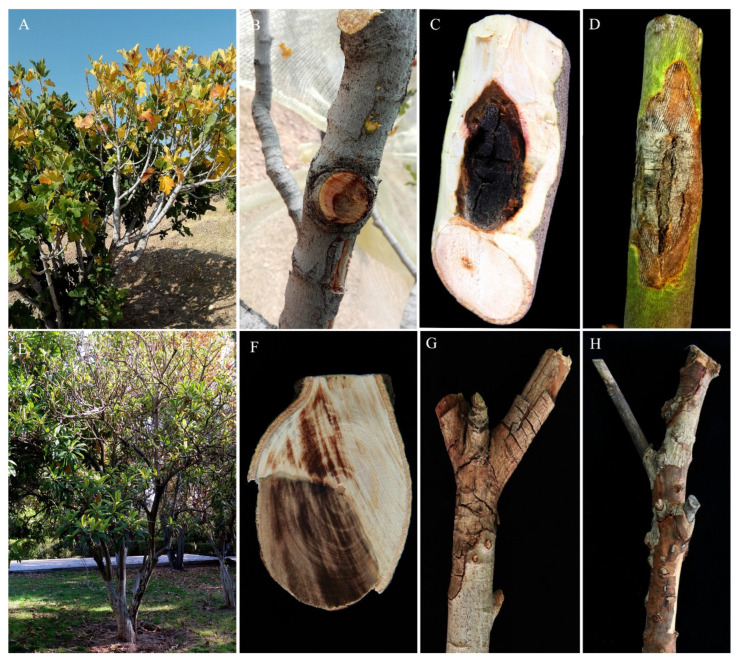
Cankers and twig dieback caused by *Stilbocrea banihashemiana* sp. nov. on main stem and branches of fig ((**A**,**B**): cv. Sabz; (**C**): cv. Siah; (**D**): cv. Pouzdonbali) and loquat (**E**–**H**) trees in Fars Province, Iran. (**A**,**E**): External symptoms, including leaves yellowing, defoliation, and branch dieback. (**B**,**F**): Internal symptoms, including brown to dark brown discoloration of wood tissues appearing as wedge-shaped necrosis in cross-sections of infected stems. (**C**,**D**): Discolored, reddish-brown, sunken, elongated cankers with intersecting longitudinal and transverse bark crackings on the main stem and branches of infected fig trees. (**G**,**H**): Dieback symptoms resulting from the expansion of lesions girdling the stems of loquat trees.

Surveys of fig and loquat orchards in the major cultivation districts of Fars Province revealed symptoms of tree decline, including stem cankers and twig dieback, were widespread. They were both external and internal (Figure 1A–H). Cankers were observed on the main stem, branches, and twigs of trees of different fig cultivars, including ‘Sabz’ (Figure 1B), ‘Siah’ (Figure 1C), ‘Pouzdonbali’ (Figure 1D). Externally, on fig trees, cankers appeared as sunken, elongated, fusiform lesions with marginal bark cracks and sometimes surrounded by a cicatricial callus (Figure 1A–D). On infected loquat trees, discolored, flaked, necrotic areas on twigs, limbs, or trunks were the predominant symptoms (Figure 1G,H). Cankers led to limb blight and twig dieback, leaf yellowing, and defoliation on both fig and loquat trees (Figure 1A,E). The internal symptoms included brown to dark brown discoloration of wood that, in cross-sections, appeared as wedge-shaped necrosis (Figure 1B,F). 

From the sampling in Estahban, Darab, Firuzabad, Jahrom, Kazerun, Khafr, Neyriz, and Shiraz counties (Fars Province), 35 isolates out of a total of 355 recovered from symptomatic fig trees, and 8 out of 77 recovered from loquat trees, respectively, were identified as a *Bionectriaceae* taxon based on morphological and molecular characteristics (Table 2). 

### 3.2. Molecular Identification and Phylogenetic Analyses

**Figure 2:**
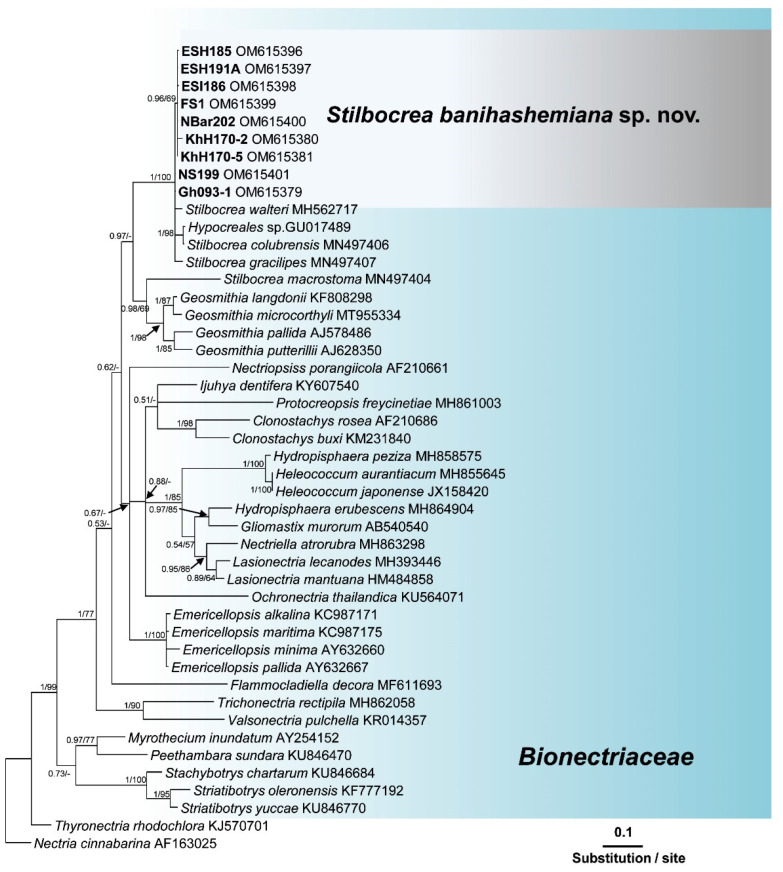
Phylogenetic position of *Stilbocrea banihashemiana* sp. nov. from infected fig and loquat trees sampled in Fars Province, Iran within *Bionectriaceae*: relationships among 35 *Bionectriaceae* members based on Bayesian analysis of ITS (internal transcribed spacers 1 and 2 and 5.8S gene of rDNA) sequences. Isolates in bold were sequenced in the present study. Numbers on the nodes are Bayesian posterior probability values (BI–PP) followed by maximum likelihood bootstrap values (ML–BS). Full supported branches (BI–PP = 1/ML–BS = 100). The tree was rooted to two *Nectriaceae* members *Nectria cinnabarina* and *Thyronectria rhodochlora*. FS1 = ex-type = CBS 148864 Isolates recovered from infected fig, and loquat trees in Iran are indicated in **bold**. Arrows represent the exact position of supporting values in the phylogenetic tree.

**Figure 3:**
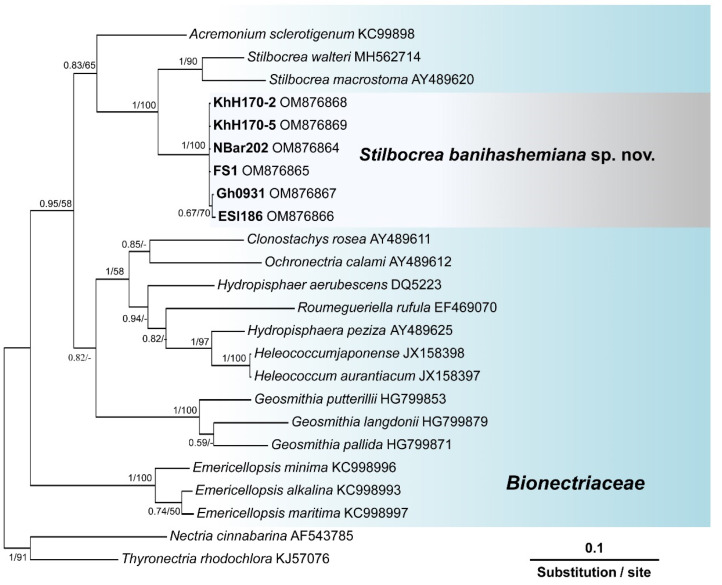
Phylogenetic position of *Stilbocrea banihashemiana* sp. nov. from infected fig and loquat trees sampled in Fars Province, Iran within *Bionectriaceae*: relationships among 16 *Bionectriaceae* members based on Bayesian analysis of *tef1* (translation elongation factor 1-α) sequences. Isolates in bold were sequenced in the present study. Numbers on the nodes are Bayesian posterior probability values (BI–PP) followed by maximum likelihood bootstrap values (ML–BS) and fully supported branches (BI–PP = 1/ML–BS = 100). The tree was rooted to *Nectria cinnabarina* (*Necriaceae*). FS1 = ex-type = CBS 148864. Isolates recovered from infected fig, and loquat trees in Iran are indicated in **bold**.

**Figure 4:**
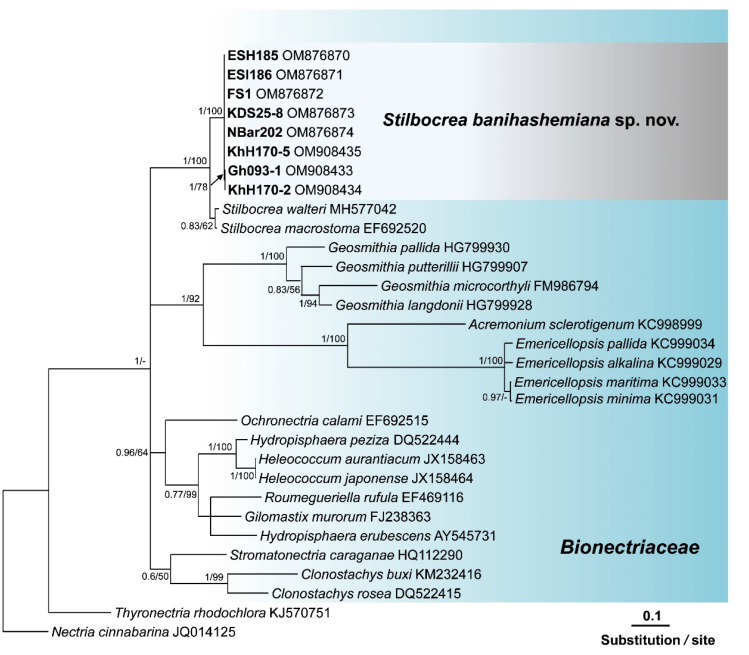
Phylogenetic position of *Stilbocrea banihashemiana* sp. nov. from infected fig and loquat trees sampled in Fars Province, Iran within *Bionectriaceae*: relationships among 21 *Bionectriaceae* members based on Bayesian analysis of *rpb2* (the second largest subunit of RNA polymerase II) sequences. Isolates in bold were sequenced in the present study. Numbers on the nodes are Bayesian posterior probability values (BI–PP) followed by maximum likelihood bootstrap values (ML–BS), Full supported branches (BI–PP = 1/ML–BS = 100). The tree was rooted to two *Nectriaceae* members, *Nectria cinnabarina* and *Thyronectria rhodochlora*¸ FS1 = ex-type = CBS 148864. Isolates recovered from infected fig, and loquat trees in Iran are indicated in **bold**. Arrow represents the exact position of supporting values in the phylogenetic tree.

**Figure 5:**
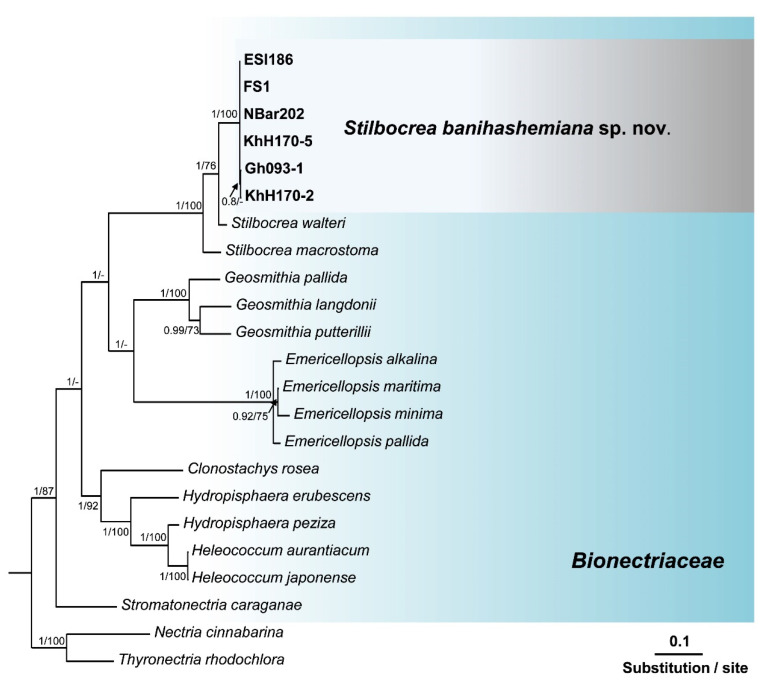
Phylogenetic position of *Stilbocrea banihashemiana* sp. nov. from infected fig and loquat trees sampled in Fars Province, Iran within *Bionectriaceae*: relationships among 15 *Bionectriaceae* members based on Bayesian analysis of multigene genealogies of ITS (internal transcribed spacers 1 and 2 and 5.8S gene of rDNA), *tef1* (translation elongation factor 1-α) and *rpb2* (the second largest subunit of RNA polymerase II) sequences. Isolates in bold were sequenced in the present study. Numbers on the nodes are Bayesian posterior probability values (BI–PP) followed by maximum likelihood bootstrap values (ML–BS) and fully supported branches (BI–PP = 1/ML–BS = 100). The tree was rooted to two *Nectriaceae* members *Nectria cinnabarina* and *Thyronectria rhodochlora*; FS1 = ex-type = CBS 148864. Isolates recovered from infected fig, and loquat trees in Iran are indicated in **bold**. Arrows represent the exact position of supporting values in the phylogenetic tree.

Search in GenBank using the Basic Local Alignment Search Tool (BLAST) showed that the ITS sequences of isolates of this unknown *Bionectriaceae* taxon, recovered from fig and loquat trees, had a similarity of 97.42% with *Hypocreales* sp. [strain KH00223, GenBank accession No. GU017492 [35]], and 97.23% similarity with *Stilbocrea walteri* Voglmayr & Jaklitsch [strain CBS 144627, GenBank accession No. NR160063 [16]]. The *ef1* sequences of these isolates also had a similarity of 93.80% with *S. walteri* [strain NQI GenBank accession No. MH562714 [16]] and their *rpb2* sequences had a similarity of 94.47% with *S. walteri* [strain NQI GenBank accession No. MH577042 [16]], and a 93.8% similarity with *Stilbocrea macrostoma* (Berk. & M.A. Curtis) Höhn [strain CBS 114375 GenBank accession No. EF692520 [36]]. The aligned datasets for ITS, *tef1*, and *rpb2* genes consisted of 538, 1287, and 994 characters, respectively. In the phylogenetic tree of three individual genes (ITS, *tef1*, and *rpb2*) and their concatenated combination, six *Bionectriaceae* isolates recovered from infected fig and loquat clustered in a well-supported monophyletic group in *the Stilbocrea* clade of *Bionectriaceae* tree (Figure 2, Figure 3, Figure 4 and Figure 5) in close vicinity of *S. walteri*. This new clade had high bootstrap support (1.00 for ML) and high posterior probability (100% for Bayesian) in both analyses. Based on the multigene phylogeny and morphology, the new lineage was proposed here as a new species, *Stilbocrea banihashemiana* sp. nov. 

To distinguish *S. banihashemiana* sp. nov. from its sister taxa, including *S. walteri*, and *S. macrostoma* (clustered in the same clade), the inter- and intraspecific variation nucleotides were detected manually (Supplementary Table S2). *Stilbocrea banihashemiana* sp. nov. aligned datasets contained 538 base pairs (bp) for ITS, 1287 bp for *tef1* and 994 bp for *rpb2*. This new lineage differed from *S. walteri* and *S. macrostoma* at 15 and 87 variable nucleotide sites in the ITS regain, 81 and 59 variable nucleotide sites in the *tef1* gene, and 55, and 50 variable nucleotide sites in the *rpb2* gen, respectively. In total, of the 2794 nucleotide characters included in the aligned three genes (ITS, *tef1*, and *rpb2*), only nine variable nucleotide sites were detected within *S. banihashemiana* sp. nov. isolates (Table 3). 

#### Taxonomy

***Stilbocrea banihashemiana*** Z. Bolboli, B. Tavakolian & Mostowf. **sp. nov**.

MycoBank 843365

*Typification*: Iran. Fars Province: Firuzabad, (28°49′.198″ N−52°33′.396″ E), isolated from the trunk of *Ficus carica*, 18 November 2020, *Z. Bolboli*, CBS 148864 (holotype) stored in a metabolically inactive state, Herbarium Westerdijk Fungal Biodiversity Institute (CBS; Utrecht, The Netherlands); FS1 = CBS 148864, ex-holotype cultures; GenBank: ITS = OM615399; *rpb2* = OM876872; *tef1* = OM876865. 

Etymology: In honor of Professor Zia Banihashemi, emeritus professor of Shiraz University, Iran, who reported the fig canker from Iran for the first time. 

**Figure 6:**
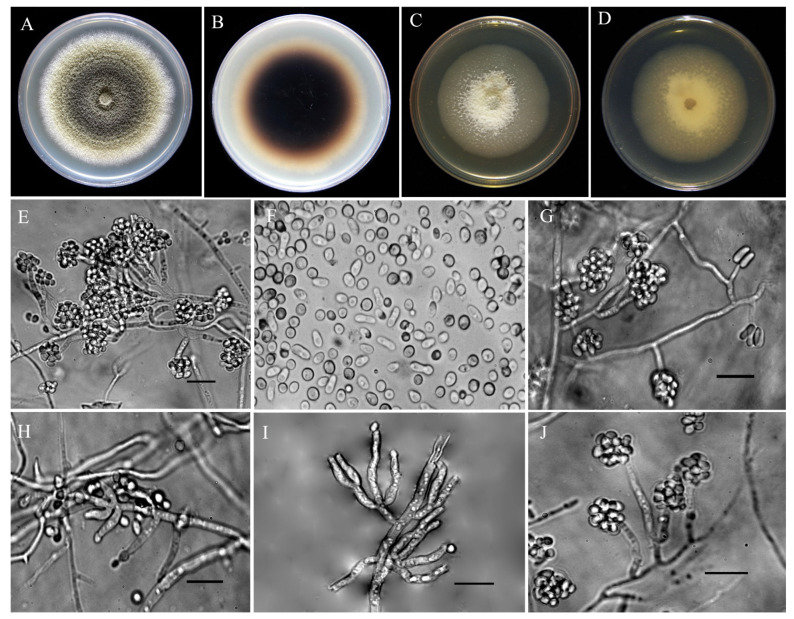
Cultural and asexual morphological characteristics of *Stilbocrea banihashemiana* sp. nov. from infected fig and loquat trees in Fars Province, Iran. (**A**,**B**): Colonies on PDA after 14 days at 25 °C under a 12-h photoperiod, obverse and reverse side view, respectively. (**C**,**D**): Colonies on MEA after 14 days at 25 °C under a 12-h photoperiod obverse and reverse side view, respectively. (**E**): Abundant globose conidia produced by phialides and long-branched conidiophores. (**F**): Small, smooth, hyaline, and unicellular conidia, in two different forms globose and allantoid. (**G**,**J**): Comparison of short and long phialides, respectively. (**H**,**I**): Long, branched, and lageniform to cylindrical phialides that formed terminally and laterally on conidiophores. Bar = 10 μm.

*Description*: Conidia abundant, small, smooth hyaline unicellular, in two different forms globose and allantoid (Figure 6F). The allantoid (4.5–7.8) × (2.12–2.79) μm (av. 5.57 ± 1.20 × 2.38 ± 0.29 μm) and globose conidia (2.9–3.56) × (2.77–3.10) μm (av. 3.28 ± 0.28 × 2.59 ± 0.61 μm). Phialides formed terminally and laterally, abundant on aerial mycelium, branched and verticillioid, cylindrical to lageniform (Figure 6E,G–J) (4.5–7.8) × (3.27–2.06) μm (av. 9.50 ± 1.80 × 2.53 ± 0.29 μm). 

Cardinal temperatures for growth: Minimum 15 °C, maximum 35 °C, optimum 30 °C.

Colonies on PDA were cottony, aerial mycelium was white at first and then from dark herbago green [37] (p. 69) to olivaceous gray [37] (p. 121) at the center, olivaceous buff at the middle ring [37] (p. 69), and with edge white (Figure 6A,B). Slow-growing colonies on PDA, with a radial growth rate of 1.25–4.2 mm/d (av. 2.88 ± 0.96 mm/d) at 25 °C, reaching 40.32 mm diam in 14 days at 25 °C under a 12-h photoperiod. On MEA, first, creamy-white colonies formed internally and slightly superficially, then white aerial mycelium was observed in the center and scattered around. Over time, the milky colonies faded and the white aerial mycelium turned olive green (Figure 6C,D). On MEA radial growth rate was 1.16–1.72 mm/d (av. 1.43 ± 0.17 mm/d) at 25 °C under a 12-h photoperiod. Colonies reached 20.02 mm diam in 14 days at 25 °C.

*Other specimens were examined (paratypes).* Isolate NBar202-1: Iran, Fars Province: Neyriz (29°08′.777″ N–54°17′.480″ E) from the branch of *Ficus carica* cv. Baranjir NBar202, 23 October 2019, *Z. Bolboli,* GenBank: ITS = OM615400; *tef1* = OM876864; *rpb2* = OM876874. Isolate ESi186: Iran, Fars Province: Estahban (29°06′.873″N–54°04′.563″E) from the trunk of *Ficus carica* cv. Siah, 23 October 2019, *Z. Bolboli,* GenBank: ITS = OM615398; *tef1* = OM876866; *rpb2* = OM876871. Isolate Gh093-1: Iran, Fars Province: Shiraz (29°40′.582″ N–52°28′.553″ E) from the branch of *Eriobotrya japonica*, 22 April 2019, *B. Tavakolian*, GenBank: ITS = OM615380; *tef1* = OM876867; *rpb2* = OM908433. Isolate KhH170-2: Iran, Fars Province: Khafr (28°59′.053″ N–53°12′.299″ E) from the branch of *Eriobotrya japonica* 1 October 2019, *B. Tavakolian*, GenBank: ITS = OM615380; *tef1* = OM876868; *rpb2* = OM908434. Isolate KhH170-5: Iran, Fars Province: Khafr (28°59′.053″ N–53°12′.299″ E) from the branch of *Eriobotrya japonica*, 1 October 2019, *B. Tavakolian*, GenBank: ITS = OM615381; *tef1* = OM876869; *rpb2* = OM908435.

*Notes:* This species belongs to the *Stilbocrea* clade of the phylogenetic tree of *Bionectriaceae* Voglmyr & Jaklitsch [16] in the proximity of *S. walteri* (Figure 2, Figure 3, Figure 4 and Figure 5). *Stilbocrea banihashemiana* sp. nov. differentiates from *S. walteri* and *S*. *macrostoma* by its two different types of globose and allantoid conidia, branched, verticillioid, cylindrical to lageniform phialides and its unique sequences of nuclear genes. Isolates were recovered from various sites in Fars Province of Iran. 

### 3.3. Pathogenicity Tests

**Figure 7:**
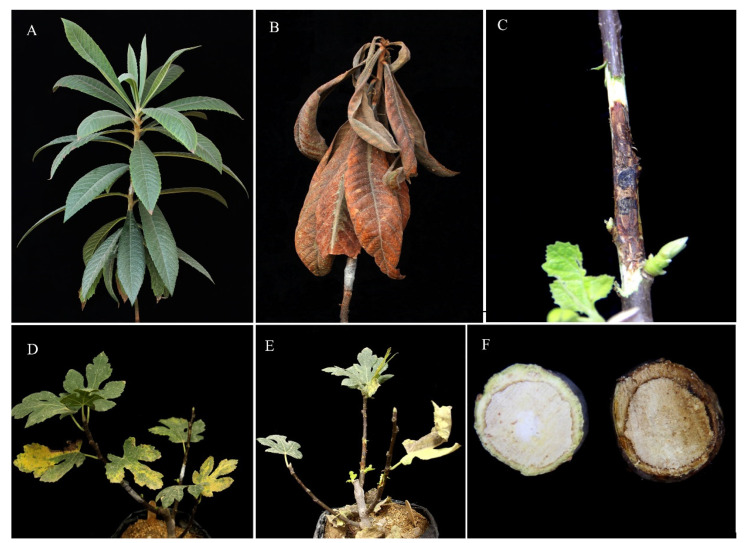
External and internal symptoms caused by *Stilbocrea banihashemiana* sp. nov. on artificially inoculated one-year-old saplings of fig and loquat. (**A**): Healthy loquat sapling (control)? (**B**): Wilting and dieback symptoms were observed 30 days after inoculation on a one-year-old loquat sapling. (**C**): Typical canker, discoloration, and necrotic lesion on a fig sapling. (**D**,**E**): External symptoms, including leaf yellowing (**D**) and defoliation (**E**), on a fig sapling 30 days after artificial inoculation. (**F**): Transerve section of a fig stem with symptoms of dieback.

**Figure 8:**
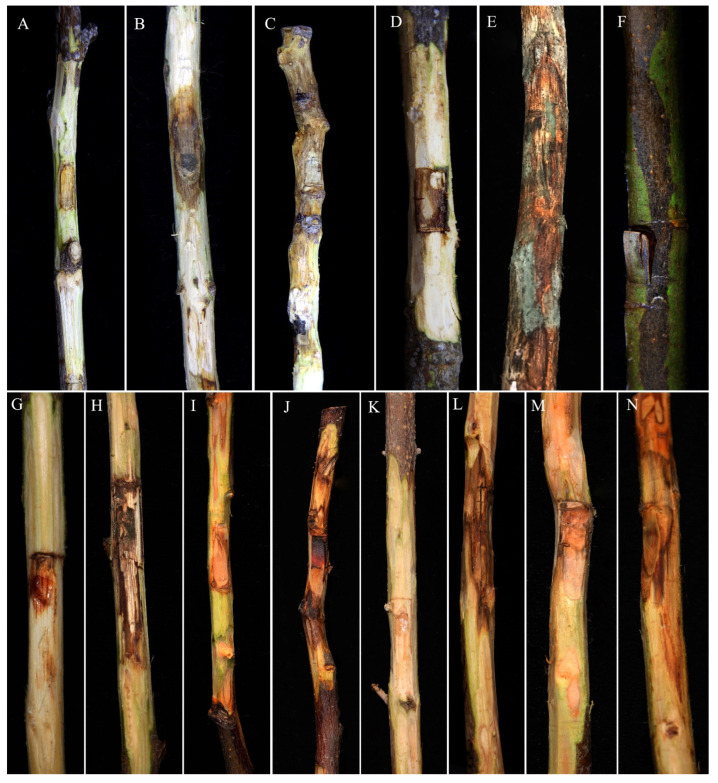
Lesions induced by *Stilbocrea banihashemiana* sp. nov. artificially inoculated on detached stems of fig, loquat, and other fruit trees compared with controls 21 days after inoculation. (**A**): Healthy *Ficus carica* cv. Sabz control, (**B**): Infected *F. carica* cv. Sabz; (**C**): Infected *F. carica* cv. Siah; (**D**): Healthy loquat; (**E**): infected loquat; (**F**): One side discoloration on infected loquat; (**G**): Healthy almond; (**H**): Infected almond; (**I**): Healthy apple; (**J**): Infected apple; (**K**): Healthy olive; (**L**): Infected olive; (**M**): Healthy quince; (**N**): Infected quince.

In pathogenicity tests on detached shoots and one-year-old fig and loquat saplings, isolates of *S. banihashemiana* caused different types of symptoms such as cankers, bark flaking, wood discoloration, twig dieback, decline, and growth reduction. In particular, on loquat saplings, they induced dieback, decline, leaf yellowing, and defoliation (Figure 7A,B). On detached stems of both fig and loquat, necrotic lesions developed upwards and downwards from the inoculation point (Figure 8A–F). Internal symptoms consisted of discolored vascular tissues that became reddish-brown to dark brown and wedge-shaped, deep wood necroses visible in cross-sections of stems, eventually leading to cambium death (Figure 7F). Both external and internal symptoms resulted in the rapid and progressive death of detached shoots at seven dpi. The primary symptoms in inoculated loquat saplings were wilting, defoliation, dieback, decline, and, finally, death of the whole sapling (Figure 7A,B). On fig saplings, isolates of *S. banihashemiana* induced cankers at first (Figure 7C) and nine months after inoculation, yellowing, and defoliation were also observed. Dieback and mortality were not observed on inoculated fig saplings (Figure 7D,E) up to 12 months after inoculation. 

In pathogenicity tests aimed at evaluating their potential host range, *S. banihashemiana* isolates were pathogenic to all tested fruit trees, including almond, olive, apple, and quince (Figure 8G–N), except pomegranate. *Stilbocrea banihashemiana* was re-isolated from infected symptomatic tissues of detached stems and saplings of fig and loquat, as well as branches of other artificially inoculated fruit trees.

## 4. Discussion

The widespread presence and high incidence of stem cankers and twig dieback associated with tree decline in fruit tree orchards in southern Iran, particularly on fig and loquat, prompted us to identify the fungal pathogens involved in this disease. So far, several ascomycetous fungi have been reported as causal agents of cankers and twig dieback on common fig and other plant species in the genus *Ficus* worldwide, including *Phomopsis cinerascens* (Sacc.) Traverso (Syn. *Diaporthe cinerascens* Sacc.), *Neofusicoccum parvum* (Pennycook & Samuels) Crous, Slippers & A.J.L. Phillips, *Lasidodiplodia theobromae* (Pat.) Griffon & Mauble, *Neoscytalidium dimidiatum* (Penz.) Crous & Slippers and *Ceratocystis ficicola* Kajitani & Masuya [5,6,10,38,39,40,41,42]. Some of these species, in particular *L. theobromae* and *N. parvum*, both in the *Botryosphaeriaceae* family, are cosmopolitan and very polyphagous pathogens, often reported as causal agents of perennial stem cankers on woody plants [41,43]. Moreover, members of *Botryosphaeriaceae* were reported to be responsible for limb cankers in loquat [7,8]. During the survey of orchards in southern Iran, fungi of *Nectriaceae* and *Didymellaceae* families were also recovered from declining fig and loquat trees, and most of them proved to be pathogenic [44]. However, this is the first report of a species of the *Bionectriaceae* family as the causal agent of trunk cankers and stem blights of fig and loquat. Morphological analyses and multiple gene phylogenetic analysis of nuclear loci (ITS, *rpb2*, and *tef1*) revealed all these isolates recovered from cankers and stems with symptoms of dieback belonged to a new species, which here has been described formally and named *Stilbocrea banihashemiana* sp. nov. Previous studies reported two other species of *Stilbocrea*, *S. walteri* and *S. macrostoma*, in Iran as pathogens of citrus and oak, respectively [17,18]. 

According to the phylogenetic analysis of three genes (ITS, *rpb2*, and *tef1*) and their concatenated combination, the *S. banihashemiana* sp. nov. isolates recovered from fig and loquat trees clustered in a monophyletic group in the *Stilbocrea* clade of *Bionectriaceae* phylogenetic tree [16]. Bayesian inference and maximum likelihood analyses showed that protein-coding genes (*rpb2* and *tef1*), mostly *tef1*, have sufficient discriminatory power to differentiate *Bionectriaceae* members, particularly *Stilbocrea* species. The *S. banihashemiana* sp. nov. appeared to be a sister taxon of *S. walteri* in Bayesian and maximum likelihood trees but differed from *S. walteri* at 15, 55, and 81 variable nucleotide sites in the ITS region, *rpb2*, and *tef1* genes, respectively. We observed a low diversity within this new lineage so that of the 2794 nucleotides characters included in the aligned three genetic regions (ITS, *rpb2*, and *tef1*), only nine variable nucleotide sites were detected.

*Stilbocrea banihashemiana* sp. nov. and *S. walteri* have some morphological characteristics in common, such as the lack of stilbella-like asexual structure and production of simple phialides in pure culture [16]. Conversely, other *Stilbocrea* species, e.g., *S. colubrensis,* and *S. macrostoma* produce synnemata in their asexual phase [15]. *Stilbocrea banihashemiana* sp. nov. differs from *S. walteri* and other *Stilbocrea* species by producing two morphologically distinct types of conidia, globose and cylindrical, which formed on short and long phialides, respectively. By contrast, *S. walteri* has only cylindrical conidia on simple short phialides. According to Voglmyr & Jaklitsch [16], the morphology of sexual and asexual reproductive structures is insufficient to distinguish different species within *Bionectriaceae* and *Nectriaceae*. Synnematous and stilbella-like asexual structures are also produced in the *Nectriaceae*, e.g., in *Nectria pseudotrichia* Berk. & M.A. Curtis [45], and acremonium-like structures (a structure in *Acremonium* species in *Bionectriaceae*) may be found in other unrelated families of the *Sordariomycetes* [46]. On the other hand, apart from *Geosmithia* and *Clonostachys* species that have been widely studied, for most *Bionectriaceae* taxa, no sequence data are available even for the ITS rDNA regions. Therefore, presently *Bionectriaceae* species identification is more challenging than for other taxonomic groups of ascomycetous fungi.

Pathogenicity evaluation of *S. banihashemiana* isolates fulfilled Koch’s postulates indicating this species is able to cause trunk and branch cankers, limb blight, and wood discoloration in fig and twig dieback in loquat. It was recovered consistently from symptomatic trees and is one of the most common ascomycetous fungi responsible for fig and loquat tree decline in southern Iran. Consequently, the decline of fig and loquat trees observed in southern Iran may be regarded as a disease with a complex etiology and, in this respect, it is similar to grapevine trunk diseases (GTDs), one of the major concerns for viticulture worldwide [47,48]. During field surveys, we observed fig cankers showed four distinct *facies* that were tentatively designated as types A, B, C, and D, respectively. Type A cankers included trunk lesions with zonations and were demonstrated to be induced by infections of *Diaporthe cinerascens* (syn. *Phomopsis cinerascens*) [5]. Type B cankers, originating from the crown of the trees and developing upward, were mostly caused by *Nectriaceae* species (Z. Bolboli and R. Mostowfizadeh-Ghalamfarsa, unpublished data). Type C cankers were extended, sunken lesions on the trunks, like those shown in the Results section (see Figure 1C,D). Type D cankers were characterized by bark crackings as well as discolored and dead areas on the trunk and main branches, like the canker shown in the Results Seciton (see Figure 1A). This study indicates that *S. banihashemiana* sp. nov. isolates can induce types C and D cankers on fig trees. Moreover, the external *facies* of cankers correlated with differences in the type of internal wood discoloration in transverse sections. Transverse sections of types C and D cankers always showed a wedge-shaped internal wood discoloration, whereas in other types of cankers, necroses were crescent-shaped, irregular-shaped, or disk-shaped. Should these differences be confirmed on a larger scale and under different environmental conditions, the type of canker could be used as a diagnostic criterion for the preliminary identification of the causal agents.

To our knowledge, this is the first report of a *Stilbocrea* species infecting fig and loquat worldwide. Consistently with our results, in a previous study, isolates of *S. macrostoma*, a closely related species, induced dieback symptoms on inoculated two-year-old oak trees [18]. In pathogenicity tests on heterologous host plants, *S. banihashemiana* isolates were also pathogenic to almond, olive, apple, and quince, suggesting very likely this new fungal species has a broader host range than just fig and loquat. The ability to infect heterologous hosts has epidemiological relevance as it implies the risk of both cross-infection between different plant species and accidental introduction of pathogen inoculum into commercial crops through alternate host plants, as already hypothesized for several other polyphagous fungus and oomycete plant pathogens [41,49,50,51].

The survey of fig and loquat orchards in southern Iran during 2019–2021 revealed *S. banihashemiana* was commonly associated with symptoms of stem cankers, twig dieback, and internal wood discoloration leading to tree decline. In previous studies, a high incidence of tree decline incited by fungi infecting stems and twigs was imputed to several factors, including a local abundance of fungal inoculum and environmental conditions predisposing the tree to the infection, such as frost, drought, water, and heat stresses [41,52,53,54]. Moreover, recently it has been highlighted a correlation between the presence of fungi associated with GTDs and the origin of propagation material, corroborating the hypothesis that fungi causing stem cankers and twig dieback in commercial orchards originate from the nursery and an endophytic lifestyle favors their spread through asymptomatic plants [41,55,56,57,58,59]. All these epidemiological aspects would deserve to be further investigated. On the basis of current knowledge, a sustainable management strategy for tree decline should include measures aimed at both preventing or mitigating the effect of predisposing environmental factors and reducing the amount of inoculum in the orchard, such as proper management of the irrigation to avoid water stress, use of windbreaks to shelter the trees from winds and of shading nets to protect branches and limbs from sun scalds as well as pruning to remove withered twigs and branches.

## Figures and Tables

**Table 1:** PCR conditions used in this study.

Gene ^1^	Number of Cycles	Initial Denaturation	Denaturation	Annealing	Extension	Final Extension
ITS	30	95 (120) ^2^	95 (45)	64 (45)	72 (45)	72 (600)
*tef1*	35	95 (120)	95 (120)	65 (60)	72 (50)	72 (600)
*rpb2*	35	95 (120)	95 (120)	66.5 (60) ^3^	72 (45)	72 (600)

^1^ ITS = internal transcribed spacers 1 and 2 and 5.8 S gene of rDNA; *tef1* = translation elongation factor 1-α; *rpb2 =* the second largest subunit of RNA polymerase II. ^2^ Temperature, °C (time, s). ^3^ For loquat isolates: 56 (80).

**Table 2:** List of *Stilbocrea banihashemiana* sp. nov. isolates recovered from infected fig (*Ficus carica*) and loquat (*Eriobotrya japonica*) trees in Fars Province of Iran.

Isolate	Location	Host	Date of Sampling	Latitude	Longitude
ES184-1	Estahban	*Ficus carica* cv. Sabz	23 October 2019	29°06′.794″ N	54°04′.533″ E
ES184-2	Estahban	*F. carica* cv. Sabz	23 October 2019	29°06′.794″ N	54°04′.533″ E
ES184-3	Estahban	*F. carica* cv Sabz	23 October 2019	29°06′.794″ N	54°04′.533″ E
ESH185-1	Estahban	*F. carica* cv. Shah Anjeer	23 October 2019	29°06′.852″ N	54°04′.596″ E
ESH185-2	Estahban	*F. carica* cv. Shah Anjeer	23 October 2019	29°06′.852″ N	54°04′.596″ E
ESi186-1	Estahban	*F. carica* cv. Siah	23 October 2019	29°06′.873″ N	54°04′.563″ E
ESi186-2	Estahban	*F. carica* cv. Siah	23 October 2019	29°06′.873″ N	54°04′.563″ E
ESi187-1	Estahban	*F. carica* cv. Siah	23 October 2019	29°06′.873″ N	54°04′.584″ E
ESi187-2	Estahban	*F. carica* cv. Siah	23 October 2019	29°06′.873″ N	54°04′.584″ E
ESi189-1	Estahban	*F. carica* cv. Siah	23 October 2019	29°06′.856″ N	54°04′.583″ E
ESH191A-1	Estahban	*F. carica* cv. Shah Anjeer	23 October 2019	29°06′.852″ N	54°04′.567″ E
ESH191A-2	Estahban	*F. carica* cv. Shah Anjeer	23 October 2019	29°06′.852″ N	54°04′.567″ E
NS195-1	Neyriz	*F. carica* cv. Sabz	23 October 2019	29°08′.777″ N	54°17′.480″ E
NS198-1	Neyriz	*F. carica* cv. Sabz	23 October 2019	29°08′.760″ N	54°17′.514″ E
NS198-2	Neyriz	*F. carica* cv. Sabz	23 October 2019	29°08′.760″ N	54°17′.514″ E
NS199-1	Neyriz	*F. carica* cv. Sabz	23 October 2019	29°08′.755″ N	54°17′.500″ E
NS199-2	Neyriz	*F. carica* cv. Sabz	23 October 2019	29°08′.755″ N	54°17′.503″ E
NS200-1	Neyriz	*F. carica* cv. Sabz	23 October 2019	29°08′.747″ N	54°17′.503″ E
NS200-2	Neyriz	*F. carica* cv. Sabz	23 October 2019	29°08′.747″ N	54°17′.503″ E
NS201-1	Neyriz	*F. carica* cv. Sabz	23 October 2019	29°08′.730″ N	54°17′.512″ E
NS201-2	Neyriz	*F. carica* cv. Sabz	23 October 2019	29°08′.730″ N	54°17′.512″ E
NS202	Neyriz	*F. carica* cv. Sabz	23 October 2019	29°08′.759″ N	54°17′.470″ E
NBar202-1	Neyriz	*F. carica* cv. Puzdonbali	23 October 2019	29°08′.777″ N	54°17′.480″ E
NBar202-2	Neyriz	*F. carica* cv. Puzdonbali	23 October 2019	29°08′.777″ N	54°17′.480″ E
NSiDrj-1	Neyriz	*F. carica* cv. Siah	18 November 2020	29°08′.777″ N	54°17′.480″ E
ESi217-1	Estahban	*F. carica* cv. Siah	18 November 2020	29°06′.794″ N	54°04′.471″ E
ECH218-1	Estahban	*F. carica* cv. Barg Chenary	18 November 2020	29°06′.858″ N	54°04′.549″ E
ECH218-2	Estahban	*F. carica* cv. Barg Chenary	18 November 2020	29°06′.858″ N	54°04′.549″ E
KDS22-3	Kazerun	*F. carica* cv. Sabz	8 January2019	29°49′.482″ N	51°47′.688″ E
KDS25-8	Kazerun	*F. carica* cv. Sabz	1 January2019	29°49′.503″ N	51°47′.633″ E
FS1 *	Firuzabad	*F. carica* cv. Sabz	18 November 2020	28°49′.198″ N	52°33′.396″ E
FS2	Firuzabad	*F. cacrica* cv. Sabz	18 November 2020	28°49′.198″ N	52°33′.396″ E
DMS1	Darab	*F. carica* cv. Sabz	3 March 2021	29°56′.446″ N	53°18′.129″ E
DMS2	Darab	*F. carica* cv. Sabz	3 March 2021	29°56′.446″ N	53°18′.129″ E
Gh093-1	Shiraz-	*Eryobotria japonica*	1 October 2019	29°40′.582″ N	52°28′.553″ E
KhH170-1	Khafr	*E. japonica*	1 October 2019	28°59′.053″ N	53°12′.299″ E
KhH170-2	Khafr	*E. japonica*	1 October 2019	28°59′.053″ N	53°12′.299″ E
KhH170-3	Khafr	*E. japonica*	1 October 2019	28°59′.053″ N	53°12′.299″ E
KhH170-4	Khafr	*E. japonica*	1 October 2019	28°59′.053″ N	53°12′.299″ E
KhH170-5	Khafr	*E. japonica*	1 October 2019	28°59′.053″ N	53°12′.299″ E
KhH170-6	Khafr	*E. japonica*	1 October 2019	28°59′.053″ N	53°12′.299″ E
KhH170-7	Khafr	*E. japonica*	1 October 2019	28°59′.053″ N	53°12′.299″ E

* ex-type = CBS 148864.

**Table 3:** List of *Stilbocrea banihashemiana* sp. nov. and their GenBank accession numbers.

Isolate	GenBank Accession Number			Isolate	GenBank Accession Number		
	ITS	*tef1*	*rpb2*		ITS	*tef1*	*rpb2*
ESH185-1	OM615396	N/A	OM876870	KDS25-8	N/A	N/A	OM876873
ESi186-1	OM615398	OM876866	OM876871	FS1 *	OM615399	OM876865	OM876872
ESH191A-1	OM615397	N/A	N/A	Gh093-1	OM615379	OM876867	OM908433
NS199-1	OM615401	N/A	N/A	KhH170-2	OM615380	OM876868	OM908434
NBar202-1	OM615400	OM876864	OM876874	KhH170-5	OM615381	OM876869	OM908435

* ex-type= CBS 148864.

## Data Availability

Not applicable.

## Supplementary Materials

The following supporting information can be downloaded at: https://www.mdpi.com/article/10.3390/jof8070694/s1, Supplementary Table S1: DNA accession numbers of *Bionectriaceae* taxa used in the phylogenetic analyses in this study; Supplementary Table S2: Base pair (bp) differences across ITS, *tef1*, and *rpb2* sequences showing the inter- and intraspecific variation of *Stilbocrea banihashemiana* sp. nov., and other related species, including *S. walteri* and *S. macrostoma*.
