# Supplementary material for: Stilbocrea banihashemiana sp. nov. a New Fungal Pathogen Causing Stem Cankers and Twig Dieback of Fruit Trees
Source: J Fungi (Basel). 2022 Jun 30;8(7):694. doi: 10.3390/jof8070694 (PMC9319130; doi:10.3390/jof8070694)
Supplement: Supplementary file 1 [file jof-08-00694-s001.zip › jof-1781588-supplementary.pdf]

Supplementary Table S1. DNA accession numbers of *Bionectriaceae* taxa used in the phylogenetic analyses in this study.

Taxon	GenBank accession number		
	**ITS**	***rpb2***	***tef1***
Acremonium sclerotigenum	-	KC998999	KC998988
Clonostachys buxi	KM231840	KM232416	-
Clonostachys rosea	AF210686	DQ522415	AY489611
Emericellopsis alkalina	KC987171	KC999029	KC998993
Emericellopsis maritima	KC987175	KC999033	KC998997
Emericellopsis minima	AY632660	KC999031	KC998996
Emericellopsis pallida	AY632667	KC999034	KC998998
Flammocladiella decora	MF611693	-	-
Geosmithia langdonii	KF808298	HG799928	HG799879
Geosmithia microcorthyli	MT955334	FM986794	-
Geosmithia pallida	AJ578486	HG799930	HG799871
Geosmithia putterillii	AJ628350	HG799907	HG799853
Gliomastix murorum	AB540540	FJ238363	-
Heleococcum aurantiacum	MH855645	JX158463	JX158397
Heleococcum japonense	JX158420	JX158464	JX158398
Hydropisphaera erubescens	MH864904	AY545731	DQ522344
Hydropisphaera peziza	MH858575	DQ522444	AY489625
Hypocreales sp.	GU017489	-	-
Ijuhya dentifera	KY607540	-	-
Lasionectria lecanodes	MH393446	-	-
Lasionectria mantuana	HM484858	-	-
Myrothecium inundatum	AY254152	-	-
Nectria cinnabarina	AF163025	JQ014125	AF543785
Nectriella atrorubra	MH863298	-	-
Nectriopsis sporangiicola	AF210661	-	-
Ochronectria calami	-	EF692515	AY489612
Ochronectria thailandica	KU564071	-	-
Peethambara sundara	KU846470	KU846508	-
Protocreopsis freycinetiae	MH861003	-	-
Roumegueriella rufula	-	EF469116	EF469070
Stachybotrys chartarum	KU846684	AY489642	AY489609
Stromatonectria caraganae	MH863716	HQ112290	HQ112286
Stilbocrea colubrensis	MN497406	-	-
Stilbocrea gracilipes	MN497407	-	-
Stilbocrea macrostoma	MN497404	EF692520	AY489620
Stilbocrea walteri	MH562717	MH577042	MH562714
Striatibotrys oleronensis	KF777192	-	-
Striatibotrys yuccae	KU846770	-	-
Trichonectria rectipila	MH862058	-	-
Thyronectria rhodochlora	KJ570701	KJ570751	KJ570768
Valsonectria pulchella	KR014357	-	-
Valsonectria simpsonii	MH862742	-	-

Supplementary Table S2. Base pair (bp) differences across ITS, *tef1*, and *rpb2* sequences showing the inter- and intraspecific variation of *Stilbocrea banihashemiana* sp. nov., and other related species, including *S. walteri* and *S. macrostoma*.

Region	Alignment length (bp)	Differences *S. banihashemiana* /*S. walteri* (bp)	Differences *S. banihashemiana* /*S. macrostoma* (bp)	Differences within *S. banihashemiana* (bp)
ITS	538	15	87	0-6
tef1	1287	82	59	0-2
rpb2	994	55	50	0-1
Total	2819	152	196	9
